# Supplementary material for: Dual RNA-seq reveals large-scale non-conserved genotype × genotype-specific genetic reprograming and molecular crosstalk in the mycorrhizal symbiosis
Source: ISME J. 2019 Jan 15;13(5):1226–38. doi: 10.1038/s41396-018-0342-3 (PMC6474227; doi:10.1038/s41396-018-0342-3)
Supplement: Supplementary file 1 — Supplementary figures and notes [file 41396_2018_342_MOESM1_ESM.docx]

**Supplementary figures and notes**

**Dual RNA-seq reveals large-scale non-conserved genotype x genotype specific genetic reprograming and molecular crosstalk in the mycorrhizal symbiosis**

Ivan D. Mateus, Frédéric G. Masclaux, Consolée Aletti, Edward C. Rojas, Romain Savary, Cindy Dupuis & Ian R. Sanders

**Supplementary Figure S1: Experimental approach.** Randomly chosen fine roots containing fungal structures (shown in blue) were used for RNA extraction and library preparation.

**Supplementary Figure S2: Fungal colonization and growth response of cassava to the inoculation with *R. irregularis*. (a)** Percentage of root length colonized by *R. irregularis*. **(b)** Total plant dry weight. **(c)** Aboveground plant dry weight. **(d)** Fine root dry weight. **(e)** Bulking root dry weight. **(f)** Total belowground dry weight. **(g)** Plant height. Brown bars represent plants inoculated with DAOM197198. Blue bars represent plants inoculated with B1. Grey bars represent mock-inoculated plants. * Represents fungal treatments that displayed statistically significant differences. Statistics are given in Supplementary Table 2.

**Supplementary Figure S3: Overview of RNA-sequencing results. (a)** Barplot showing the number of sequenced gene transcripts in cassava and *R. irregularis* per sample. Colour of bars follows that of Supplementary figure 2. **(b)** Boxplots of the number of genes sequenced in the different mycorrhizal treatments and plant cultivars. **(c)** Saturation curve of genes sequenced and the numbers of reads per sample. Red colour represents DAOM197198 samples, blue colour represents B1 samples and grey represents non-mycorrhizal plants. In cassava, the number of different cassava gene transcripts sequenced was not affected by the sequencing depth of the samples and did not differ significantly among the mycorrhizal treatments, neither from the plant treatments. In *R. irregularis*, the read number did not influence the number of transcripts between inoculated treatments and there were no significant differences in the number of different transcripts among cassava cultivars.

**Supplementary Figure S4: Summary of plant transcriptional differences between cassava inoculated with isolates DAOM197198 and B1 and transcriptional differences between the two *R. irregularis* isolates on biotic stress pathways in each cassava cultivar.** **(a)** Log2 fold change in cassava gene transcription between plants inoculated with isolates DAOM197198 and B1. **(b)** Log2 fold change in *R. irregularis* gene transcription between *R. irregularis* isolates DAOM197198 and B1. Values are reported for each cassava cultivar. Functional categories were obtained by using the Mercator online tool and the figure is modified from the Mapman software output. Darker red colours represent a lower log2 fold change in treatments inoculated with isolate DAOM197198 compared to isolate B1. Darker blue colours represent higher log2 fold change in treatments inoculated with isolate DAOM197198 compared to isolate B1. White represents no fold change.

**Supplementary Figure S5: Overview of *R. irregularis* and cassava module formation and correlation to the partner organism.** WGCNA tools Hierarchical clustering and Dynamic tree cut were used to assign genes into different modules (Coloured bars). **(a)** Cassava genes differentially transcribed between the plants inoculated with the mycorrhizal isolates and the mock-inoculation (1634) were clustered into 9 modules. Different colours represent different modules. (**b)** All transcribed *R. irregularis* genes (7840) were clustered into 20 different modules represented by different colours. **c)** Correlation matrix between all the cassava modules (rows) and all the *R. irregularis* modules (columns). The correlation coefficient and its *p-value* were reported for each module-module correlation. Highly positive correlations are shaded dark blue, and highly negative correlations are shown in dark green. Downstream statistical analyses involved module-module correlations where their *p-value* was < 0.001. To control for a possible multiple-comparison bias, a threshold cut-off was set at *p* = 0.001. If we correlate 9 modules of cassava to 20 modules of *R. irregularis*, by chance we would expect 0.18 module-module correlations with this threshold.


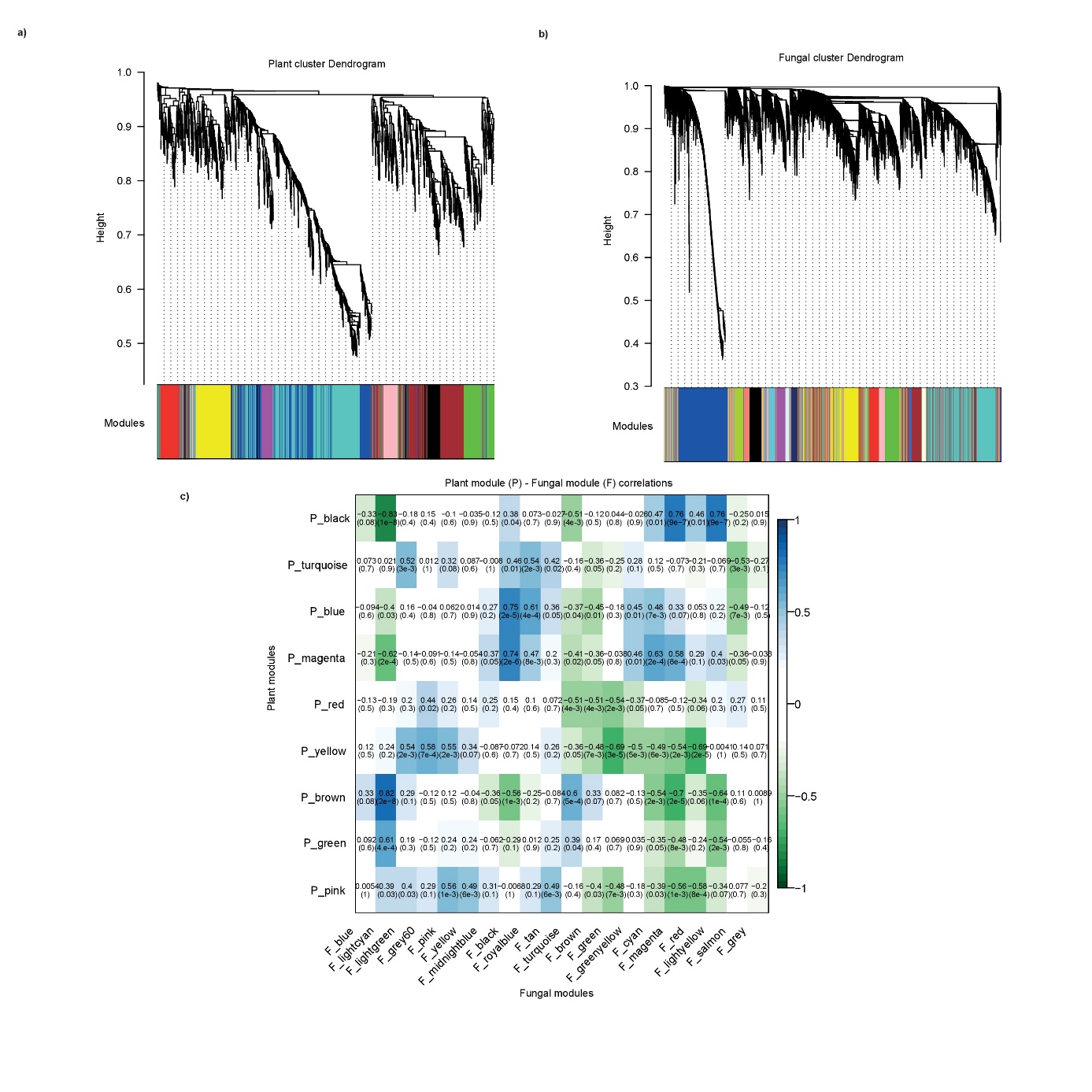


**Supplementary Figure S6: Module co-expression networks are not random correlations.** We randomly assigned each gene to the 9 cassava and 20 *R. irregularis* modules, creating modules containing random genes. Circle’s size represents the number of genes. **(a)** A random *R. irregularis* module and a random cassava module, each containing 100 randomly assigned genes, did not display any significant GO terms. On the other hand, the correlated *R. irregularis* and cassava modules displayed significant GO terms, with the exception of *R. irregularis* module F_cyan and cassava module P_green. Module colours refer to names of identified modules shown in in Supplementary Figure 5. **(b)** Network of random *R. irregularis* and cassava modules. No correlated modules were observed. Brown circles represent *R. irregularis* modules. Green circles represent cassava modules. **(c)** Correlation matrix between all the cassava random modules (rows) and all the *R. irregularis* random modules (columns). We reported the correlation coefficient and its *p-*value for each module-module correlation. Highly positive correlations are shown in dark blue, and highly negative correlations are shown in dark green. Downstream statistical analysis involved module-module correlations where was *p* ≤ 0.001. To control for a possible multiple-comparison bias, a threshold cut-off was set at *p* = 0.001. If we try to correlate 9 modules of cassava with 20 modules of *R. irregularis*, we could expect by chance that with this threshold we would find 0.18 module-module correlations. Colours refer to modules identified in Supplementary Figure 5.


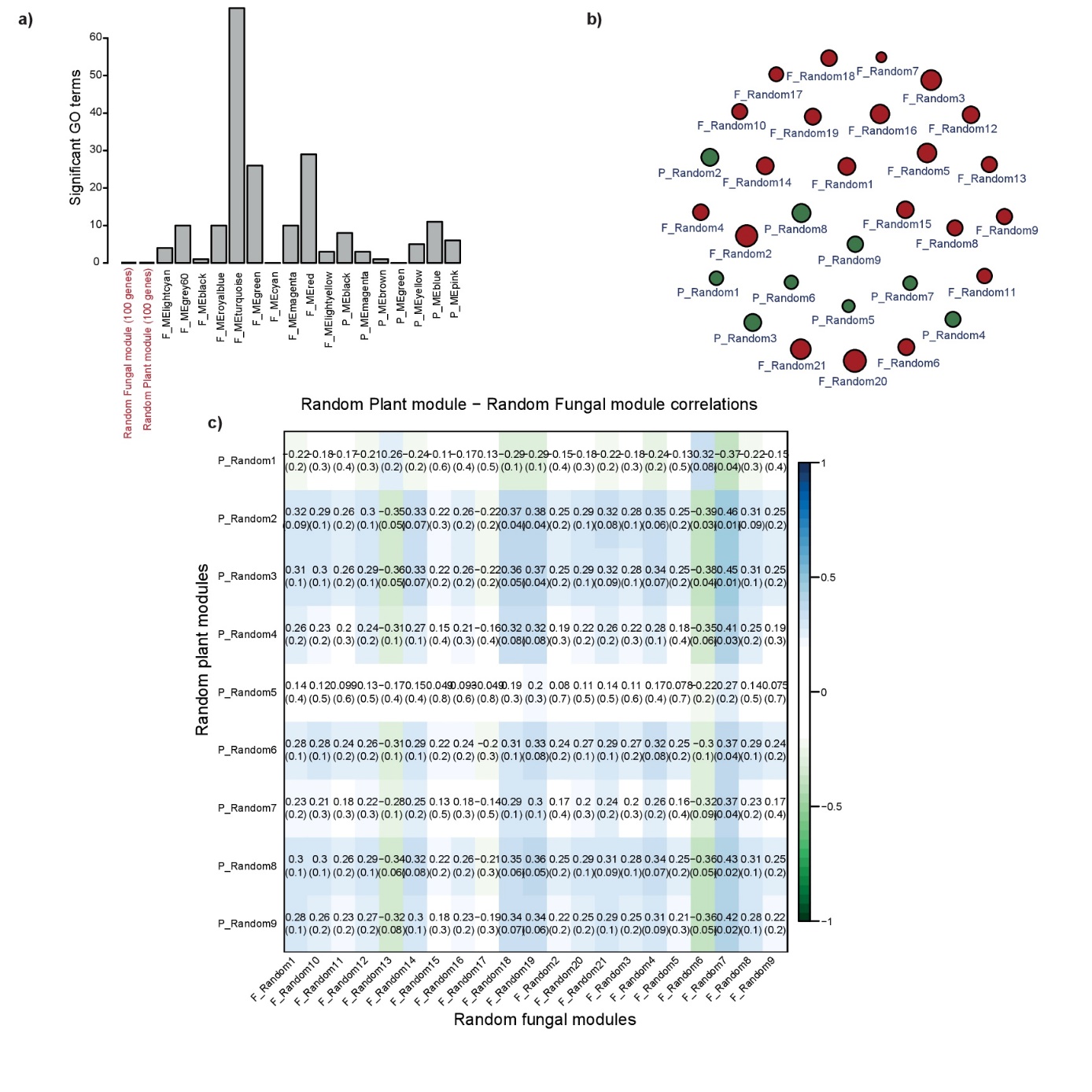


**Supplementary note S1: Greenhouse experiment.** We micro-propagated *in vitro* plants using cuttings from lateral and apical meristems of approximately 1 cm long. The cassava cultivars used in this study were originated from: Palmira, Colombia (CM6438-14, CM4574-7, CM523-7), Pivijay, Colombia (COL2215) and Belen, Brazil (BRA337). Explants were grown on MS medium with 14 h long daylight (light intensity 100 μE.m-2s-1) at 25°c in a culture chamber (Sanyo MLR-351 H). After 8 weeks of growth, plants were hardened off in greenhouse conditions (28°C, 16 h daylight and 70% RH) for four weeks. The hardening substrate was an autoclaved mixture of perlite and peat moss (1:1). Hardened plants were transplanted to final steam sterilized (100°C, 25 min) substrate; composed by perlite, moss peat, inert clay and sand (1:1:1:1) (v/v). We used *R. irregularis* isolates DAOM 197198 (originally collected in Pont Rouge, Canada) and isolate B1 (originally collected in Tänikon, Switzerland) to inoculate the plants. Spores from *in-vitro* split-plate cultures were extracted by dissolving from phytagel in a solution of citric acid (6%), and passed through a 30µm sieve. We inoculated the plants with a 10ml suspension containing 300 spores of either isolate in distilled water and the mock-inoculated treatment received 10 ml distilled water. Plants were inoculated by applying the suspension or water directly to the root zone. Following inoculation, plants were grown in greenhouse conditions for 18 weeks. We carried 15 biological replicates for each one of the 15 different treatments, summarizing 225 plants. We arranged the plants in a randomised block design in the greenhouse, where each block contained one replicate each of the different treatments. Additionally, we randomised the position of the blocks every 4 weeks in order to avoid block effects.

**Supplementary note S2: Fungal colonization measurements.** We cleared the roots with 10% KOH for 4 hrs, acidified them with HCl (1%) during 5 minutes and stained them with trypan blue (0.10% in a lactic acid-glycerol solution) overnight. Root colonization was determined using 10 replicates of each treatment by the grid line intersect method [1].

**Supplementary note S3: RNA extraction, library preparation and sequencing.** We extracted RNA from 3 replicates of each treatment of each cultivar. Approximately 150 mg of fine roots were randomly collected. The RNA was extracted from root tissue using the Maxwell plant RNA kit (Promega). We used the *TruSeq Stranded mRNA* Library Prep Kit, set B, to make the library preparations. The libraries were sequenced using Illumina Hi-Seq® 100 nucleotide paired-end sequencing in 6 separate lanes. In all steps of the RNA extraction, library preparation and sequencing, we randomized the samples in order to avoid batch effects at these steps.

**Supplementary note S4: Bioinformatic analyses**. We processed the raw reads with the script Tagcleaner.pl to trim Illumina adapters [2]. Reads were quality-filtered and trimmed using PrinSeq-lite.pl version 0.20.4 [3]. Low quality 3’-ends were trimmed and reads containing uncalled bases (N) removed. Only reads longer than 50 bp were kept for further analyses. To separate cassava and *R. irregularis* data sets, following quality control and trimming, we aligned the all remaining reads to the *Manihot esculenta* reference genome (*M. esculenta* V6.1, Phytozome V11) with a two-pass method using the STARstatic 2.4.0 aligner [4]. Then, for the cassava dataset, we extracted the aligned reads with samtools [5] (bam2fq option). For the *R. irregularis* dataset, we ensured that the sequence reads were not from cassava by using only the reads that did not map to the cassava reference genome. By doing this, we obtained two separate datasets for cassava and for *R. irregularis*. We then used the pseudo aligner kallisto [6] in order to produce count tables of the number of counts per transcript per sample. For cassava we used the online gene prediction produced for Mesculenta_V6.1. For *R. irregularis*, we produced a gene prediction of the reference genome N6 [7] with Augustus (see Gene prediction in *R. irregularis*).

**Supplementary note S5: Differential expression analysis**. After normalization by transcript length, in kallisto, we worked at the gene-level by using the tximport R function [8]. We then transformed the raw data into a DGElist object (edge R package) [9]. We filtered out the genes that contained less than 100 counts in at least 3 samples and normalized the samples by the library size using the calcNormFactors function (edge R package). We proceeded to transform the count data to logCPM and estimate the mean-variance relationship using the voom function (limma R package) [10]. Finally, we used the lmFit, ebayes and topTable functions (limma package) to fit a linear model, compute the statistics for differential transcription and extract the differentially transcribed genes between the treatments. We used the multiple comparisons FDR method as correction to detect significantly different gene transcription between the treatments (Adj.p-value < 0.05).

**Supplementary note S6: Identification of orthologous genes in *M. esculenta***. A list of core *M. esculenta* genes involved in the symbiosis was established based on Hogekamp and Küster [11], and references therein. The protein sequences coded by these genes were retrieved from the *Medicago truncatula* proteome. Orthologs were searched in the *M. esculenta* proteome with ggsearch36 (Threshold e-value 1e-10, http://faculty.virginia.edu/wrpearson/fasta/fasta36/). Orthologs were conserved when they could be unambiguously identified from other matches based on the % identity, the overlap length and the % similarity. Only “late-stage” genes were considered, which were defined as those expressed in the symbiosis after 23 days. We did not consider genes that are usually involved in the very early stages of root colonization by mycorrhizal fungi as the plants in this experiment grew for several months, following inoculation.

**Supplementary note S7: Gene prediction in *R. irregularis.*** Prediction of protein coding genes was performed with the *ab initio* gene prediction tool Augustus based on a hidden Markov model. Augustus was trained with a dataset of *Aspergillus* *sp.* proteins. The *Aspergillus* protein dataset was generated by retrieving proteins, reviewed by Swissprot, from www.uniprot.org. Scipio [12] and BLAT [13], which were then used to align the *Aspergillus* proteins with the N6 genome and to define the gene structure. The structure of the genes was used to train and optimize Augustus following Augustus instructions (http://www.molecularevolution.org/molevolfiles/exercises/augustus/training.html). We generated hints for Augustus predictions from a publicly available DAOM197198 RNA-seq dataset using STAR aligner and cufflinks. Augustus predictions with hints were performed on the repeat-masked N6 genome using the trained parameters for the species. In order to confirm the predicted proteins resulting from each gene, we performed a Blastp query to the non-redundant protein sequence (nr) database (NCBI). The annotation file of the predicted protein sequences of *R. irregularis* can be found in Supplementary data 1.

**Supplementary note S8: Gene ontology (GO) terms, functional classification and pathway diagrams.** We obtained the GO terms corresponding to each of the predicted genes of both organisms using the Blast2GO tool with standard parameters [14]. Blast was run against the NCBI-NR sequence database. We then used the R package ‘GOSeq’ to conduct a GO term enrichment analysis [15], using the “BP” ontology with a node-size threshold of 5 genes. To detect which biological processes are the more representative in each module, we took as total gene set all the sequenced genes, and performed the enrichment analysis on all the genes contained in each module. We classified each gene into a functional category by using the Mercator sequence annotation tool (<http://www.plabipd.de/portal/mercator-sequence-annotation)> with the default parameters, adding Interpro scan, ORYZA and CHLAMY parameters. The different pathway diagrams were made using MapMan 3.5.1R2 [16] providing the functional classification obtained by the Mercator sequence annotation. We provided an experimental dataset to MapMan obtained by selecting the logFC of the significantly differentially transcribed genes between the conditions.

**Supplementary notes references**

1. Giovannetti M, Mosse B. An evaluation of techniques for measuring vesicular arbuscular mycorrhizal infection in roots. *New Phytol* 1980; **84**: 489–500.

2. Schmieder R, Lim YW, Rohwer F, Edwards R. TagCleaner: Identification and removal of tag sequences from genomic and metagenomic datasets. *BMC Bioinformatics* 2010; **11**: 341.

3. Schmieder R, Edwards R. Quality control and preprocessing of metagenomic datasets. *Bioinformatics* 2011; 1–3.

4. Dobin A, Davis CA, Schlesinger F, Drenkow J, Zaleski C, Jha S, et al. STAR: Ultrafast universal RNA-seq aligner. *Bioinformatics* 2013; **29**: 15–21.

5. Li H, Handsaker B, Wysoker A, Fennell T, Ruan J, Homer N, et al. The Sequence Alignment/Map format and SAMtools. *Bioinformatics* 2009; **25**: 2078–2079.

6. Bray NL, Pimentel H, Melsted P, Pachter L. Near-optimal probabilistic RNA-seq quantification. *Nat Biotechnol* 2016; **34**: 525–527.

7. Lin K, Limpens E, Zhang Z, Ivanov S, Saunders DGO, Mu D, et al. Single nucleus genome sequencing reveals high similarity among nuclei of an endomycorrhizal fungus. *PLoS Genet* 2014; **10**: e1004078.

8. Soneson C, Love MI, Robinson MD. Differential analyses for RNA-seq: transcript-level estimates improve gene-level inferences. *F1000Research* 2015; **4**: 1521.

9. Robinson MD, McCarthy DJ, Smyth GK. edgeR: A Bioconductor package for differential expression analysis of digital gene expression data. *Bioinformatics* 2009; **26**: 139–140.

10. Smith GK. limma: Linear Models for Microarray Data. *Bioinforma Comput Biol Solut Using R Bioconductor* 2005; 397–420.

11. Hogekamp C, Küster H. A roadmap of cell-type specific gene expression during sequential stages of the arbuscular mycorrhiza symbiosis. *BMC Genomics* 2013; **14**: 306.

12. Keller O, Odronitz F, Stanke M, Kollmar M, Waack S. Scipio: using protein sequences to determine the precise exon/intron structures of genes and their orthologs in closely related species. *BMC Bioinformatics* 2008; **9**: 278.

13. Kent WJ. BLAT— The BLAST-Like Alignment Tool. *Genome Res* 2002; **12**: 656–664.

14. Götz S, Garcia-Gomez JM, Terol J, Williams TD, Nagaraj SH, Nueda MJ, et al. High-throughput functional annotation and data mining with the Blast2GO suite. *Nucleic Acids Res* 2008; **36**: 3420–3435.

15. Young MD, Wakefield MJ, Smyth GK, Oshlack A. Gene ontology analysis for RNA-seq: accounting for selection bias. *Genome Biol* 2010; **11**: R14.

16. Thimm O, Bläsing O, Gibon Y, Nagel A, Meyer S, Krüger P, et al. MAPMAN: A user-driven tool to display genomics data sets onto diagrams of metabolic pathways and other biological processes. *Plant J* 2004; **37**: 914–939.
